# A Case of Hypocomplementemic Urticarial Vasculitis Syndrome With Severe Renal and Gastrointestinal Involvement

**DOI:** 10.7759/cureus.72113

**Published:** 2024-10-22

**Authors:** Lorena Jost, Birgit M Helmchen, Michael Osthoff, Luzia Nigg Calanca

**Affiliations:** 1 Department of Internal Medicine, Kantonsspital Winterthur, Winterthur, CHE; 2 Department of Pathology and Molecular Pathology, University Hospital Zurich, Zurich, CHE; 3 Department of Nephrology, Kantonsspital Winterthur, Winterthur, CHE

**Keywords:** anti-c1q antibody, colon ischemia, huvs, hypocomplementemic urticarial vasculitis, hypocomplementemic urticarial vasculitis syndrome, impaired kidney function, low complement

## Abstract

We present a severe case of hypocomplementemic urticarial vasculitis syndrome (HUVS) and its diagnostic and therapeutic challenges. A 56-year-old male presenting with fever and impaired kidney function was diagnosed with HUVS. Before the initiated treatment was effective, he developed severe colon ischemia, and a subtotal colectomy was required. We discuss other affected organs, such as kidneys, lungs, the heart, and the skin. Pathophysiology is briefly reviewed and the difficulty of overlapping autoimmune diseases is discussed. Treatment continues to be challenging, as there is no consensus about the optimal immunosuppressive therapy.

## Introduction

Hypocomplementemic urticarial vasculitis syndrome (HUVS) is a rare disease first described in 1973 by McDuffie et al. [1]. It develops mostly in the fifth decade of life, affecting women more often than men [2]. HUVS is classified as an immune complex small vessel vasculitis [3]. Diagnostic criteria were proposed by Schwartz et al. in 1982. Two major criteria (urticaria for more than six months and hypocomplementemia) must be met in addition to at least two minor criteria (dermal venulitis, arthralgia, or arthritis, and mild glomerulonephritis, ocular involvement, recurrent abdominal pain, or elevated anti-C1q antibody concentrations) [4]. Potential organ involvement includes the skin, joints, kidneys, lungs, the gastrointestinal tract, eyes, the heart, and the nervous system [5]. Pathogenesis of the disease remains to be elucidated, but anti-C1q antibodies and complement consumption are evidently involved [6]. There is no consensus regarding the preferred treatment regimen, and various immunosuppressive therapies have been tried with varying results [7]. The treatment recommendations are therefore mainly based on case reports or case series.

## Case presentation

**Figure 1:**
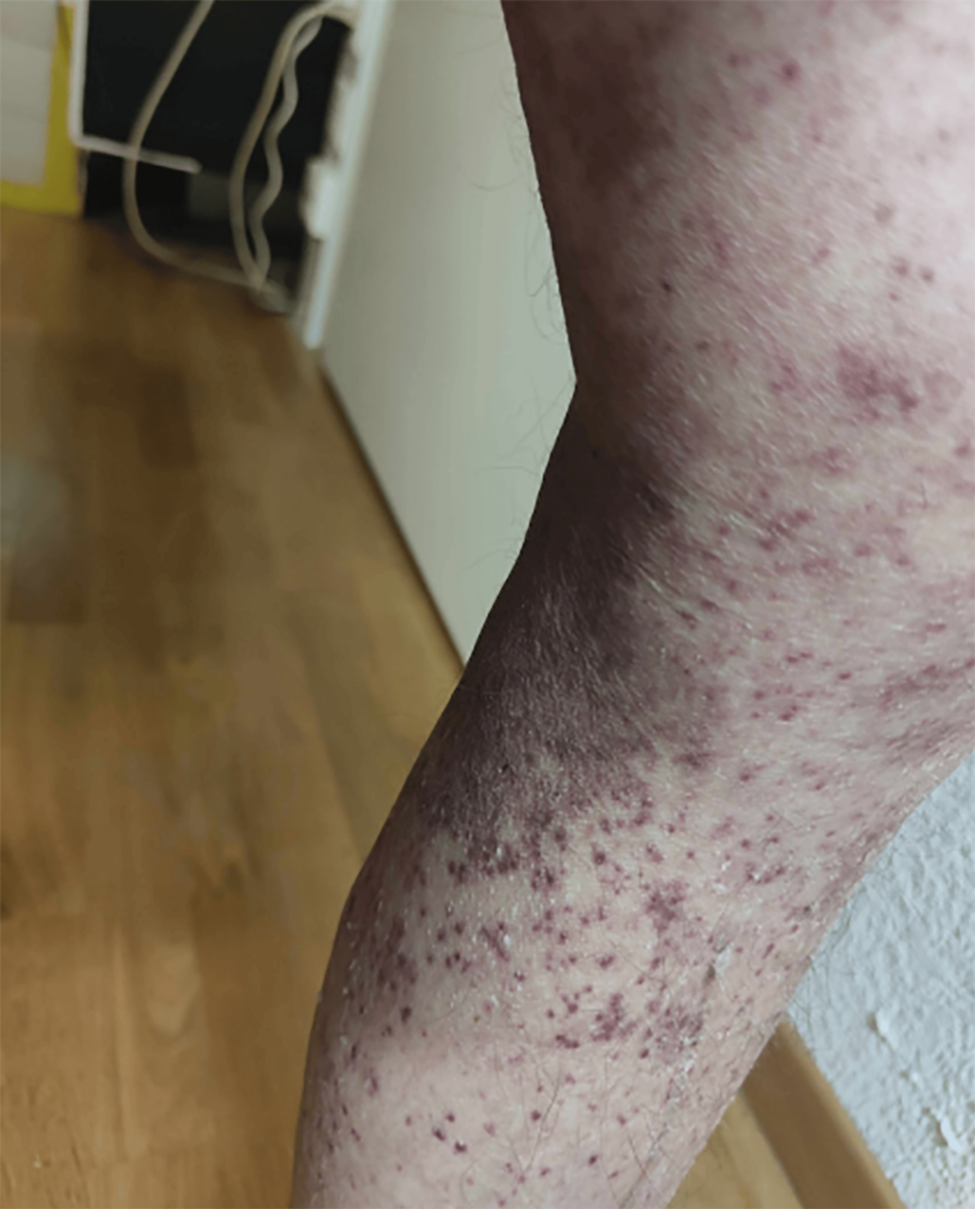
Purpura of the left lower extremity.

A 56-year-old Caucasian man was referred to our Department of Internal Medicine with fever, abdominal pain, laboratory signs of inflammation, and impaired kidney function. HUVS had been suspected in 2016 when the patient presented with wheals and arthralgia. At that time both major criteria were met as well as one minor criterion (arthralgia in hands, ankles, and knees since 2013). Skin biopsy, however, could not unequivocally demonstrate leukocytoclastic vasculitis. Histology primarily showed urticaria, though early stages of leukocytoclastic vasculitis could not be excluded. Nonetheless, the patient was initially treated with sulfasalazine, then hydroxychloroquine, followed by methotrexate, which had been suspended about a month prior to the current hospitalization because of pancytopenia. At that time the patient had been hospitalized because of cellulitis of the face. Three weeks before that the purpura of the lower extremities (Figure 1) had been treated with corticosteroids by the general physician.

Further history included former intravenous drug use, currently receiving medication-assisted treatment with morphine (though the patient admitted to additional nasal consumption of cocaine and heroin), osteoporosis, hypertensive heart disease, and spontaneously cleared hepatitis C. Family history included a brother with systemic lupus erythematosus (SLE). A physical examination revealed a tender abdomen and urticarial-like lesions at the upper arms and thighs. The laboratory investigation showed moderate normocytic anemia, moderate CRP elevation (55 mg/l, normal range < 10 mg/l), and increased serum creatinine (389 µmol/l, normal range: 64-111 µmol/l). Urinalysis revealed tubular proteinuria (albuminuria: 139 mg/g, normal range <30 mg/g; proteinuria: 1324 mg/g, normal range < 300 mg/g; and alpha-1-microglobulin/creatinine ratio: 358 mg/g, normal range < 14 mg/g), microhematuria (399/ul, normal range < 20/ul) with 15 % acanthocytes (normal range < 5%), and granular casts.

An immunological work-up (antinuclear antibodies (ANA), anti-neutrophil cytoplasmic antibodies (ANCA), cryoglobulins, rheumatoid factors, anti-cardiolipin antibodies, anti-β2 glycoprotein antibodies, anti-dsDNA, anti-Sm, anti-SS-A, anti-SS-B, anti-U1RNP, anti-Rib-P, anti-PCNA, anti-CENP-B, anti-Sc170, anti-RNA-Polymerase III, anti-fibrillarin, anti-PM-Scl, anit-Mi-2, and anti-Jo-1) was negative, except for an elevated anti-C1q antibody concentration (171 U/mL, normal range < 20 U/mL). Concentrations of complement components C3 and C4 were markedly decreased (C3: 0.4 g/l, normal range: 0.9-1.8 g/l, and C4: 0.06 g/l, normal range: 0.1-0.4 g/l). Anti-histon antibody concentration was elevated (2.7 U, normal range < 1 U), as well as anti-chromatin antibody concentration (39 E/ml, normal range < 20 E/ml). HIV and hepatitis B virus (HBV) serology tests were negative. Hepatitis C virus (HCV) RNA was not detected. Given the clinical presentation with fever, wheals, and impaired kidney function in addition to the current evidence of complement consumption and elevated anti-C1q antibody concentration, the previously suspected diagnosis of HUVS seemed likely, and further tests were indicated.

**Figure 2:**
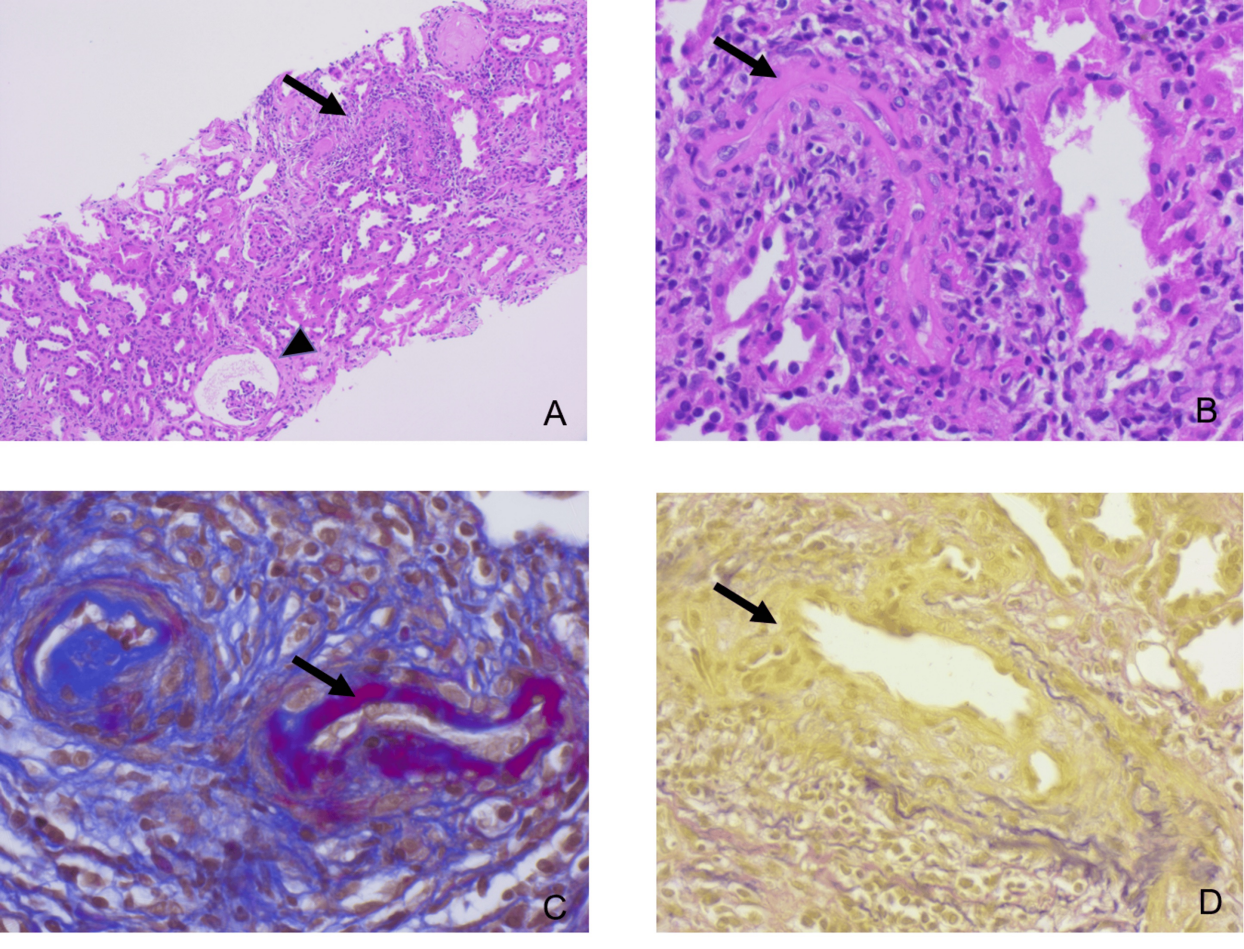
Light microscopy of the kidney biopsy cylinder. (A) Hematoxylin and eosin (H&E) stain. The long arrow shows fibrinoid necrosis of an artery with infiltration of inflammatory cells. The short arrow points to an ischemic glomerulus. Tubulointerstitial are signs of acute tubular damage and regeneration (dilation of tubular lumina, flattened epithelium, and regeneratively altered epithelial cell nuclei). (B) H&E stain. Artery with fibrinoid necrosis and infiltration of inflammatory cells (long arrow) accompanied by perivascular inflammatory cells. Notably, there are also many perivascular inflammatory cells. (C) Acid-fuchsin orange-G stain. Artery with fibrinoid necrosis (long arrow) and infiltration of inflammatory cells. (D) Elastica Van Gieson's stain. Artery with partial destruction of the lamina elastica (long arrow); the preserved lamina elastica is stained black.

**Figure 3:**
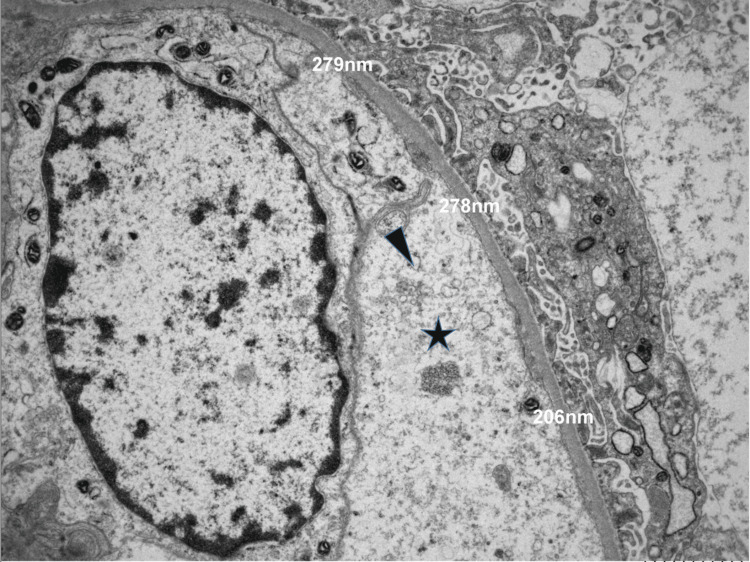
Electron microscopy of the kidney biopsy. Reticular aggregates (star) within the cytoplasm of an endothelial cell (short arrow) in a glomerular capillary loop.

A kidney biopsy was performed. Light microscopy showed no signs of glomerulonephritis. Only three of the 27 glomeruli were completely sclerosed; the remaining glomeruli had no abnormalities. The main finding was diffuse interstitial fibrosis and tubular atrophy affecting 70% of the cortex along with tubulointerstitial inflammatory cells and tubulitis as well as acute tubulointerstitial injury and regeneration. Additionally, features of vasculitis, e.g., fibrinoid necrosis of small arteries, were seen (Figure 2). Luminal IgG, but no C1q, was observed along small vessels, though immunofluorescence testing was performed not on cryopreserved specimens but on deparaffinized tissue. By electron microscopy, reticular aggregates were detected in the endothelial cytoplasm (Figure 3). These are usually a manifestation of high interferon levels and might therefore be due to HUVS in this patient.

Thus, a diagnosis of HUVS was confirmed, as two major (recurrent urticarial exanthema and hypocomplementemia) and three minor criteria (abdominal pain, arthralgia, and positive anti-C1q antibody levels) were met. The current presentation fit the diagnosis of HUVS, especially considering its manifestation and worsening after cessation of immunosuppressive therapy with methotrexate. Immunosuppressive therapy using oral prednisolone 50 mg/day and mycophenolate mofetil 1000 mg/day was initiated.

**Figure 4:**
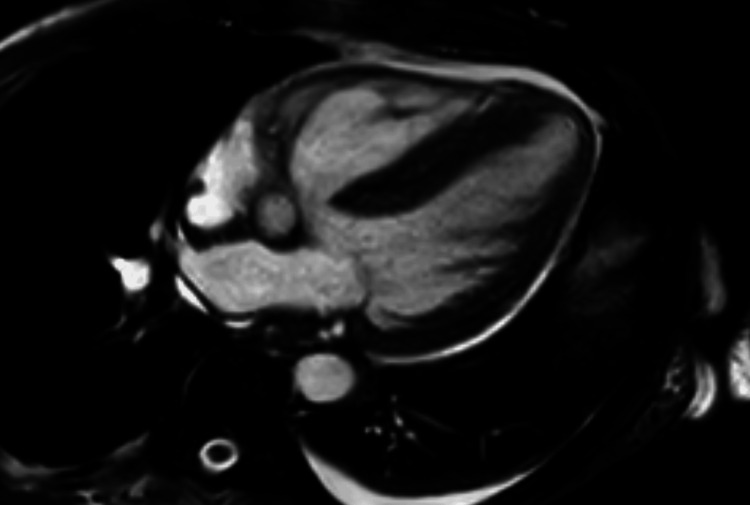
Cardiac MRI.

**Figure 5:**
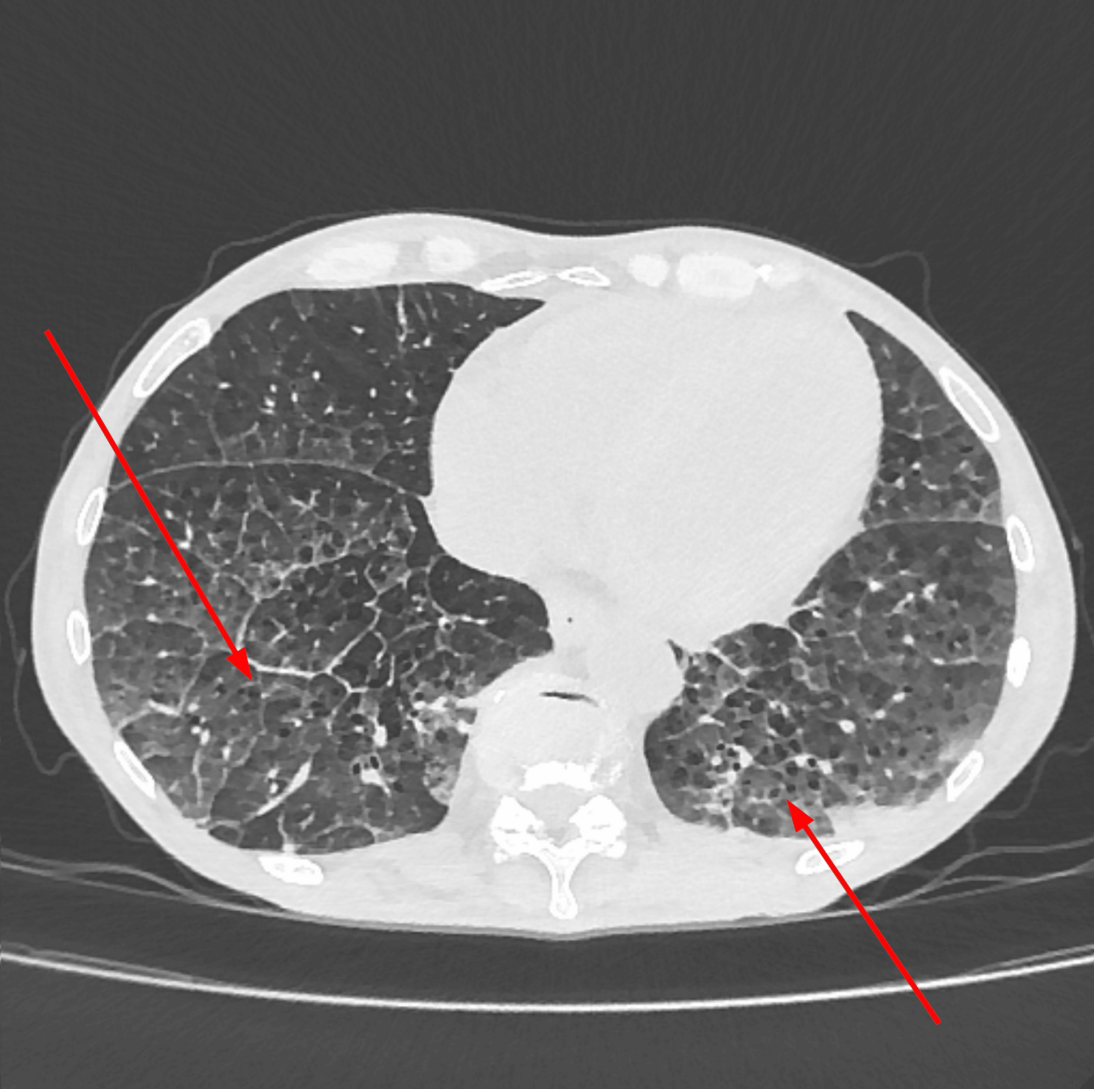
CT scan of the lungs. Crazy-paving patterns are indicated by red arrows.

The patient was also screened for other organ manifestations, where no affections of the eyes were found. However, a cardiac ultrasound revealed global hypokinesia with a reduced left ventricular ejection fraction of 40% as well as a left ventricular thrombus, with no obvious cause thereof. A cardiac MRI, performed two weeks later, revealed no active inflammation of the heart, especially no edema, fibrosis, or necrosis (Figure 4). Computed tomography (CT) of the lungs showed crazy-paving patterns with diffuse ground-glass opacities in both lower lobes. Those changes could result from vasculitis or be due to emphysema, as the patient had a history of smoking (Figure 5).

**Figure 6:**
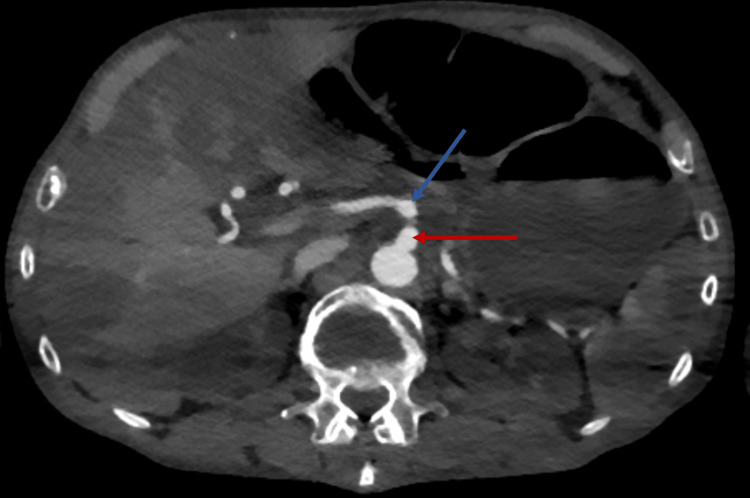
Abdominal CT scan with angiography. Blue arrow shows the superior mesenteric artery. Red arrow indicates the celiac trunk. Both arteries are filled with contrast, suggesting open vessels.

**Figure 7:**
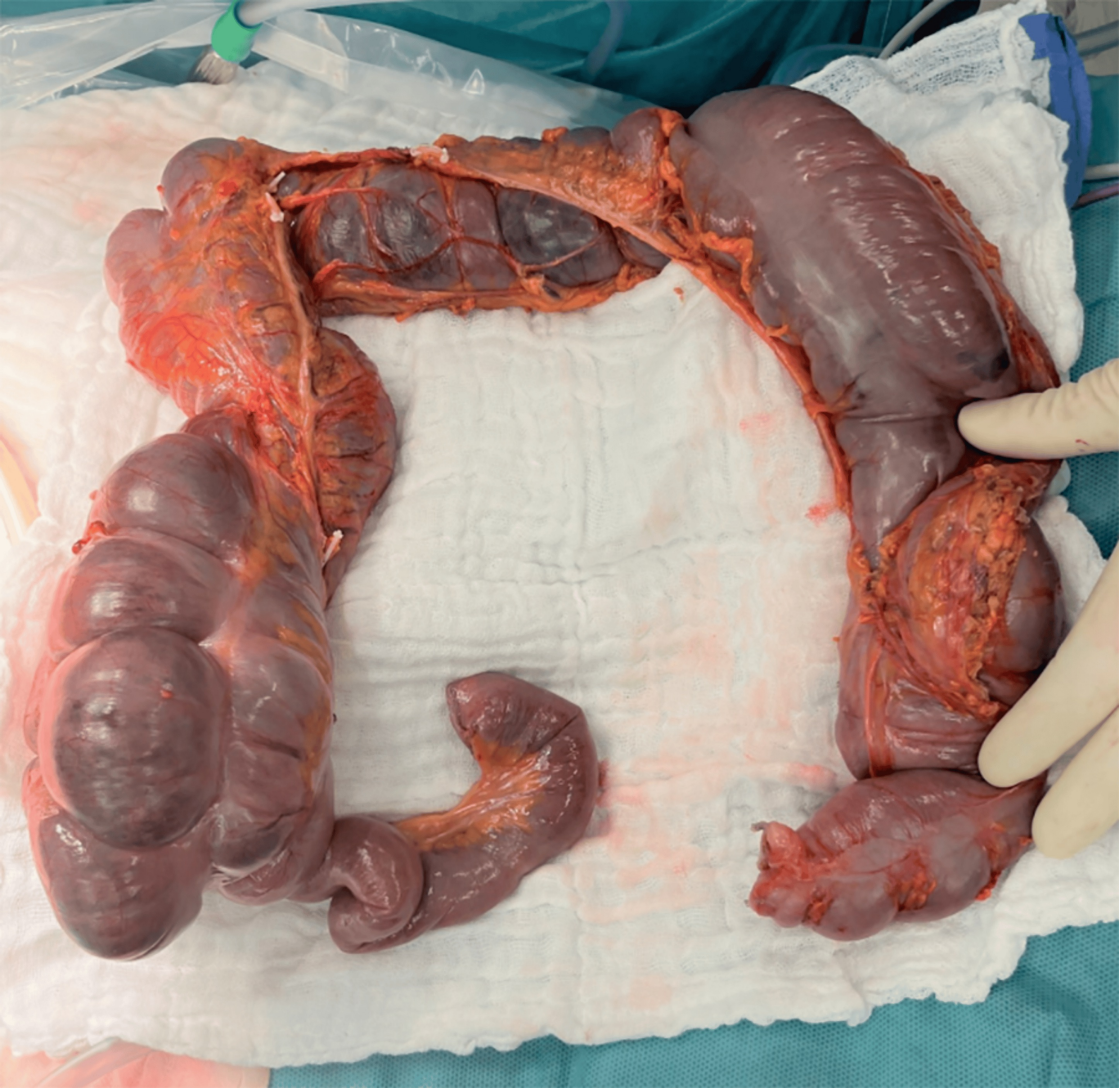
Ischemic colon resectate.

**Figure 8:**
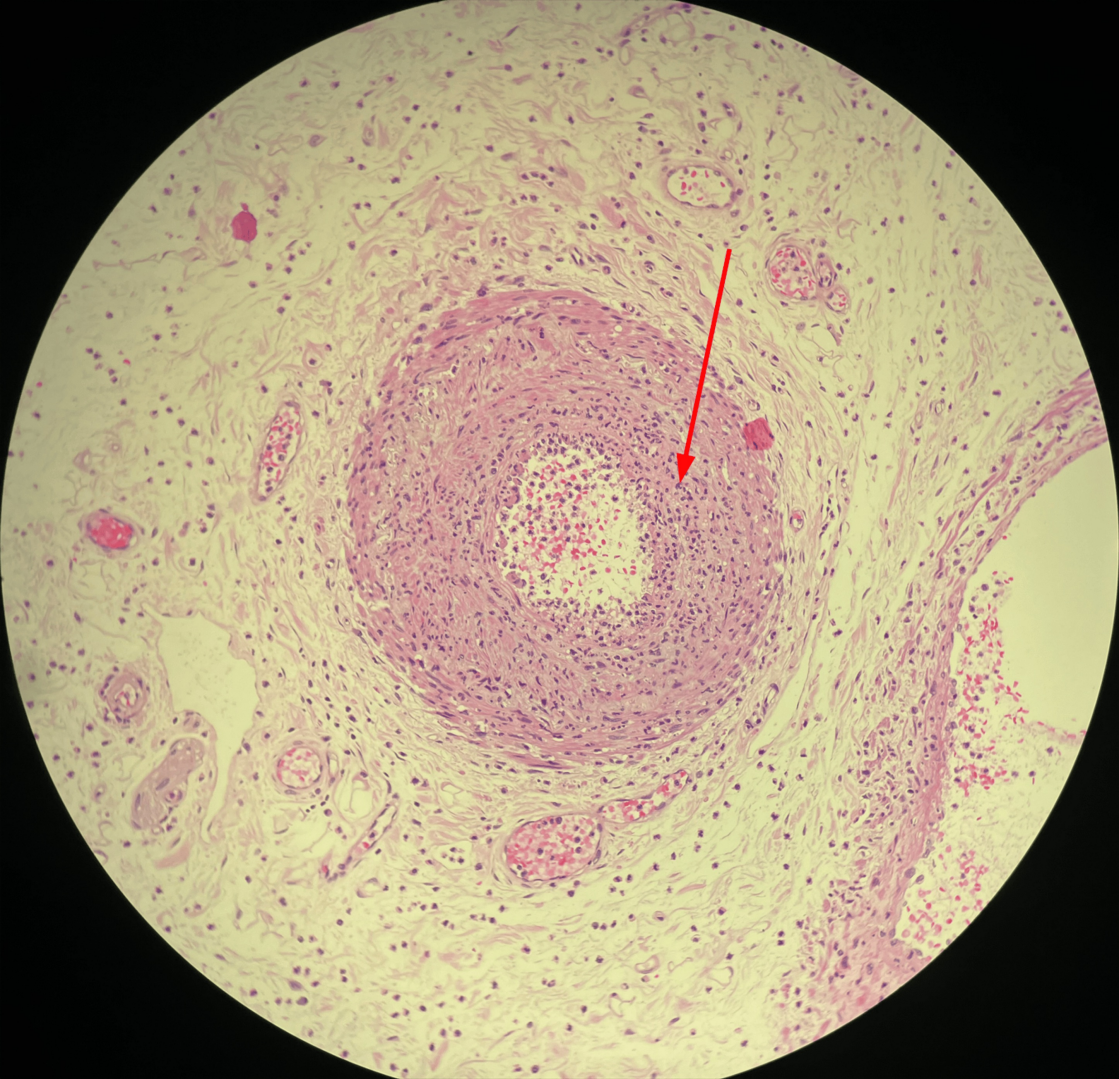
Light microscopy of the colon resectate. Hematoxylin and eosin (H&E) stain. Light microscopy showing a vessel, which has been completely infiltrated by inflammatory cells (red arrow).

Throughout the hospital stay, severe abdominal pain persisted. Ischemic lesions were observed during colonoscopy in the cecum and the ascending colon, though no clear occlusions of large or medium-sized vessels were evident on abdominal CT angiography (Figure 6). Subsequently, the patient became septic with guarding and rebound tenderness, and an emergent laparotomy was performed. Intraoperatively, almost the entire colon was found to be ischemic; thus, subtotal colectomy with terminal ileostomy was necessary. Light microscopy of the resected colon showed acute necrotizing vasculitis of submucosal vessels, i.e., fibrinoid necrosis, alongside fresh intravascular fibrin-thrombi (Figures 7, 8).

Following this severe HUVS gastrointestinal manifestation, immunosuppressive therapy was intensified with cyclophosphamide 15 mg/kg initially and 10 mg/kg three weeks later (reduced dose due to leukopenia). Subsequently, the patient received maintenance therapy with rituximab (first two doses of 1000 mg two weeks apart, then intended every six months).

**Figure 9:**
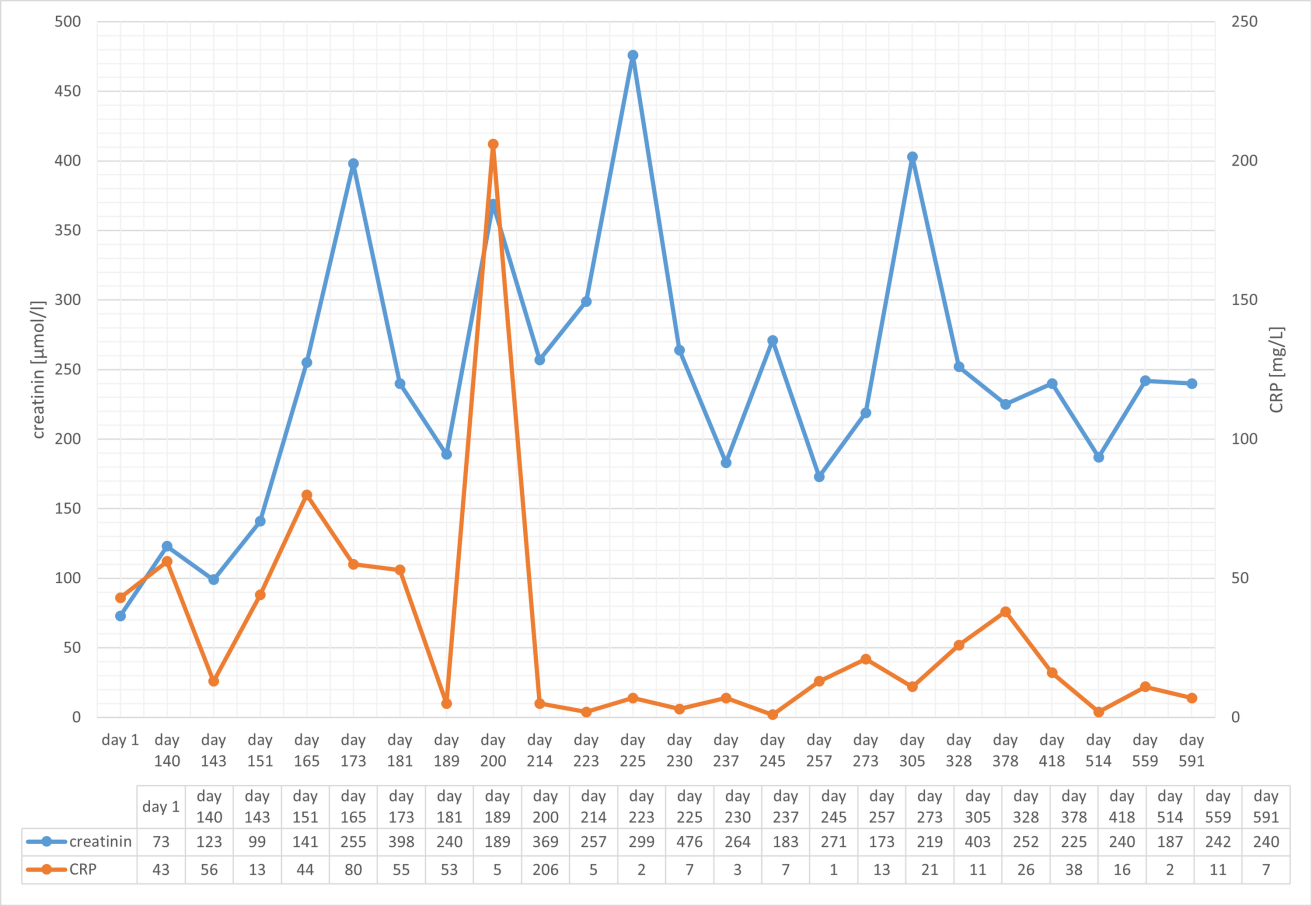
Course of creatinine and C-reactive protein (CRP). Course of creatinine (normal range: 64-111 µmol/l) and CRP (normal range < 10 mg/l). The patient was hospitalized from day 173; immunosuppressive therapy with prednisolone was started on day 182.

**Figure 10:**
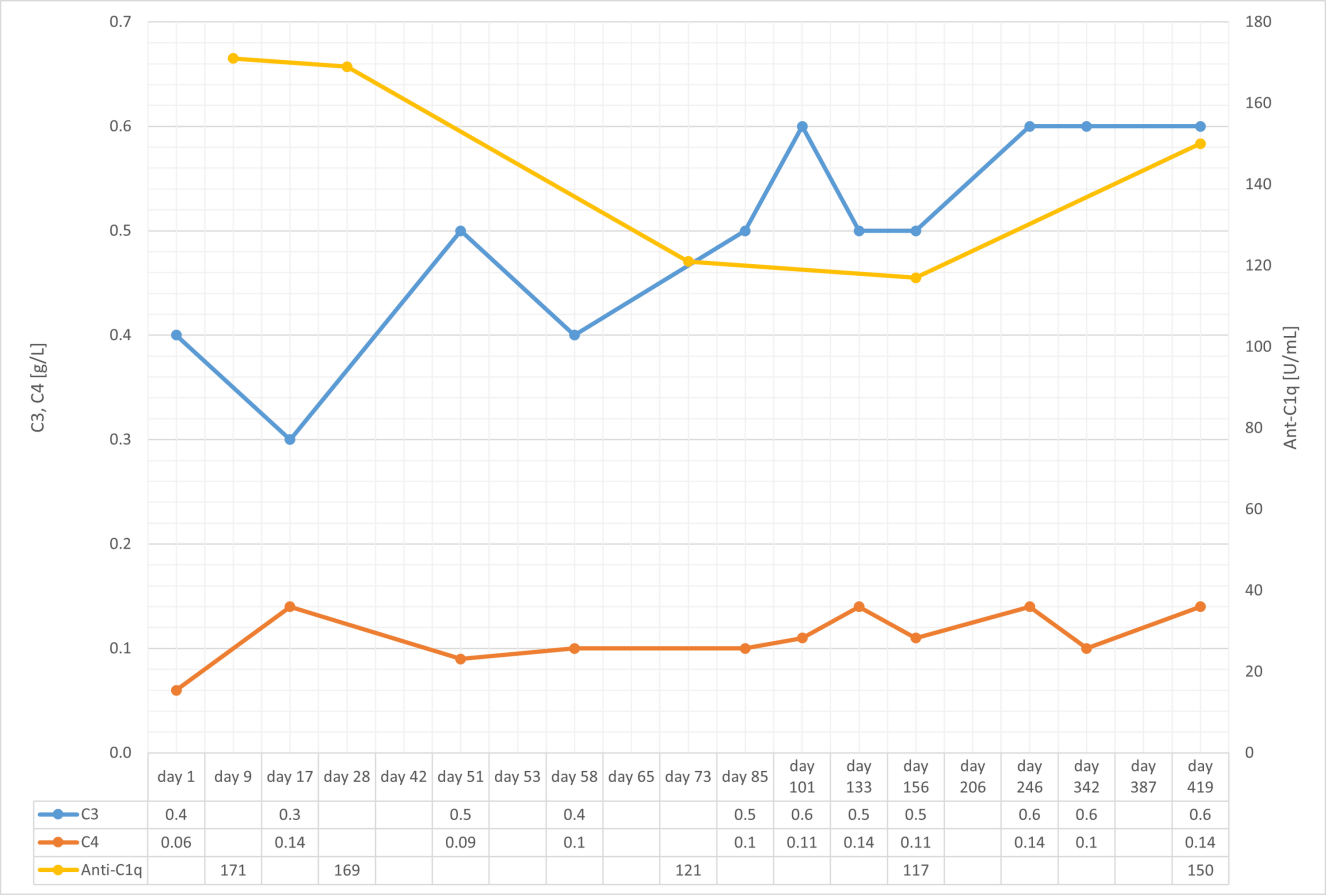
Course of C3, C4, and anti-C1q. Course of C3 (normal range: 0.9-1.8 g/l), C4 (normal range: 0.1-0.4 g/l), and anti-C1q (normal range < 20 U/mL). The patient was hospitalized on day one; immunosuppressive therapy with prednisolone was started on day 10.

At the follow-up visit 13 months after initiation of immunosuppressive therapy, renal function had partially recovered (creatinine 240 µmol/l). The patient delayed the third rituximab administration, and he then again developed arthralgia, which disappeared after rituximab was administered. Complement C4 had recovered to normal levels (0.12 g/l), while C3 was still low (0.6 g/l). Anti-C1q antibody titer first decreased to 117 U/ml at four months but increased again to 171 U/ml at 13 months. The course of laboratory results is shown in Figures 9, 10. The management of the disease in this patient was challenging, especially due to poor compliance.

## Discussion

The presented case clearly fulfills the HUVS diagnostic criteria by Schwartz et al. (Table 1) [4]. For a HUVS diagnosis, it is important to actively search for alternative diagnoses with similar presentations or overlapping features such as systemic lupus erythematosus SLE. Anti-C1q antibodies, for example, are found in 100% of HUVS patients but also in 30-48% of SLE patients, especially those with lupus nephritis (Table 2) [8]. Furthermore, 54 percent of HUVS patients have later been diagnosed with SLE [9]. There is much debate about whether HUVS is a subtype of SLE or an entirely separate entity, as well as if HUVS might progress to SLE or can be seen as a precursor to it [9-14]. Our patient did not fulfill the 2019 European League Against Rheumatism/American College of Rheumatology (EULAR/ACR) criteria for SLE, as the entry criterion for SLE, antinuclear antibodies, was negative [15]. However, the patient had elevated anti-histon and anti-chromatin antibodies, which both can be found in SLE patients. Anti-chromatin antibodies can be detected in SLE patients who don't yet show double-stranded-DNA antibodies. Anti-histon antibodies are strongly associated with medication-induced SLE. Thus, maybe our patient's condition is drug-induced. His sustained illegal drug use, with the possibility of impure substance consumption, might be a possible trigger. 

**Table 1:** Diagnostic criteria for hypocomplementemic urticarial vasculitis syndrome (HUVS). Adapted from Schwartz et al., 1982 [4]. The patient must meet both major and at least two minor criteria.

Type of criteria	Criteria
Major criteria	Chronic urticarial exanthema
	Hypocomplementemia
Minor criteria	Venulitis of the dermis
	Arthralgia or arthritis
	Glomerulonephritis
	Uveitis or episcleritis
	Recurrent abdominal pain
	Positive C1q antibodies

**Table 2:** Prevalence of laboratory findings compared for hypocomplementemic urticarial vasculitis syndrome (HUVS) and systemic lupus erythematosus (SLE). Adapted from Jara et al., 2009 [6].

Laboratory finding	HUVS, %	SLE, %
Antinuclear antibodies (ANA)	50	95
Anti-dsDNA	<5	70
Anti-Sm	0	30
Antiphospholipid antibodies	Rare	40
Anti-C1q-antibodies	100	35
Hypocomplementemia	100	62

The pathogenesis of HUVS is not fully understood, but anti-C1q antibodies seem to play an essential role. Although anti-C1q antibodies were first identified in the serum of SLE patients, as C1q precipitins [16], they have since been found in various diseases such as HUVS, Felty’s syndrome, Sjogren’s syndrome, rheumatoid vasculitis, membranoproliferative glomerulonephritis, and IgA nephropathy, as well as in some healthy individuals [17]. C1q is the first component of the classical pathway of complement activation. Its role is to support the clearance of immune complexes from tissues, as well as autoantigens generated during apoptosis. It has been shown to bind to apoptotic cells and directly initiate their phagocytosis. Furthermore, macrophages, which are exposed to bound C1q, show increased phagocytosing capacity [18]. Interestingly, homozygous deficiency of C1q was identified as the strongest genetic factor for disease susceptibility for SLE [19]. Genetic factors seem to play a role in the development of HUVS as well, as there have been documented cases of the disease in a pair of identical twins and among three siblings [20,21]. The fact that the patient in the presented case has a brother with SLE, supports a genetic susceptibility for a systemic autoimmune disease in this patient. Maybe this supposed genetic susceptibility even contributes to the severity of systemic involvement of the kidneys and the intestines. Possible genetic testing will be discussed with the patient.

The incidence of kidney involvement in HUVS is reported to range between 14% and 50% [22-24]. This wide range may be due to different screening methods (either creatinine alone or urinalysis as well) or the small number of cases. Corthier et al. found histologic kidney lesions to be various, with different patterns of glomerular, vascular, and tubulointerstitial involvements, though membranoproliferative glomerulonephritis was the most common finding [25]. Despite these different patterns in kidney biopsy, it seems reasonable that any biopsy, irrespective of location (and not skin biopsy alone), can be used to confirm a HUVS diagnosis as long as it shows leukocytoclastic, necrotizing vasculitis, the remaining diagnostic criteria are met and other autoimmune conditions have been excluded [7]. Systematic screening for kidney involvement in HUVS should be considered given its frequency and implication on morbidity and mortality. Considering that in the presented patient, creatinine levels had been rising months before kidney involvement was suspected, an earlier kidney biopsy might probably have led to an earlier start of immunosuppressive therapy and thus might have prevented severe systemic manifestation of the disease. In addition to immunosuppression, therapy may also include general progression-delaying therapies such as renin-angiotensin-aldosterone system (RAAS) blockade and a sodium-glucose cotransporter-2 (SGLT-2) inhibitor.

Gastrointestinal symptoms are present in about 30% of HUVS patients and may range from abdominal pain, nausea, vomiting, diarrhea, and ascites to hepatomegaly and splenomegaly [5,26]. To the authors' best knowledge, only one case of gastrointestinal vasculitis in a HUVS patient has previously been reported, though the course of the gastrointestinal involvement seems to have been much less severe than in the present case [27]. Intestinal vasculitis may have been more pronounced in the present case, because of the patient's cocaine consumption. Vasoconstriction due to cocaine may have worsened the already precarious perfusion of the colon affected by vasculitis.

At our patient's first presentation in 2016, skin biopsy was not distinct from simple urticaria. Clinically, urticaria resolves within 24 hours, whereas the wheals caused by urticarial vasculitis persist for longer and leave hyperpigmentation or purpura after resolution. When deeper vessels are involved in the vasculitis, angioedema may be present [5,28]. It is possible that in this case, the patient had an angioedema of the face as a manifestation of HUVS, which was misdiagnosed and treated as cellulitis. We also consider the previous occurrence of purpura of the lower extremities to be a HUVS manifestation.

Concerning possible cardiac involvement in the presented case, there has not yet been a case reported where a ventricular thrombus has been described as a manifestation of HUVS. Cardiac involvement in HUVS typically involves pericarditis, valvular abnormalities, and congestive heart failure, the latter also being present in the presented case [5,29]. It remains to be determined whether the patient's ventricular thrombus was a systemic manifestation of HUVS. It is possible that inflammation of coronary microvasculature was present but had already been suppressed by corticosteroid therapy at the time of the MRI, as no edema was visible.

If the lungs are involved, patients can present with dyspnea, coughing, hemoptysis, pleural effusion, emphysema, and chronic obstructive pulmonary disease (COPD) [4,5]. In HUVS cases, COPD is progressive and the most frequent cause of death. Smokers, such as the presented patient, typically show more severe affection of the lungs [9]. Screening for COPD has therefore been proposed as crucial and smoking cessation in all HUVS patients is recommended [30].

Studies regarding complement levels have indicated a correlation between the degree of hypocomplementemia and the severity of illness and consequently a poorer prognosis [5,11]. Anti-C1q antibodies have been proven to be an accurate marker for disease activity in SLE nephritis [31]. Accordingly, it seems reasonable that anti-C1q antibodies might also be used to evaluate disease activity in HUVS patients, although evidence supporting this approach is lacking. In the presented case, the anti-C1q antibodies have been increasing again after 13 months of immunosuppressive therapy with rituximab (which led to sustained B-cell depletion), without any objective signs of disease activity. However, the patient still experiences intermittent abdominal pain, which might reflect ongoing disease activity. Alternative causes, such as celiac disease or inflammatory bowel disease, have been excluded.

Regarding treatment, varying immunosuppressive regimens have been proposed. There are no drugs approved for HUVS therapy. Treatment recommendations are therefore mainly based on case reports or case series. Corticosteroids have been used in almost all patients and have been shown to be effective for systemic HUVS manifestations. As corticosteroids have many side effects (in the presented case, the patient already suffered from osteoporosis) and tapering is often difficult, there is a need for other effective agents. Biologicals were effective in 75% of HUVS patients, according to one review [32]. Cyclophosphamide alone or followed by rituximab should certainly be considered for life-threatening cases, cases with severe organ involvement, such as the presented case, and relapsing forms. Plasmapheresis may be used in highly active disease [5,25,33]. Its value remains unclear, however, and it must always be followed by immunosuppressive therapy.

## Conclusions

HUVS is a rare small vessel vasculitis, which may affect skin, joints, kidneys, lungs, the gastrointestinal tract, eyes, the heart, and the nervous system. It should be differentiated from other systemic diseases, especially SLE, with which it shares multiple overlapping features. HUVS patients should be screened for systemic involvement of the kidneys and lungs, as they are associated with higher morbidity and mortality. Early and aggressive immunosuppressive treatment is warranted, as shown in this case with severe gastrointestinal and kidney involvement. Further studies regarding the optimal immunosuppressive regimen are needed.
